# Intra-articular Methotrexate-Loaded Microsponge as an Adjuvant Strategy for Rheumatoid Arthritis: Localized Treatment with a Systemic Impact

**DOI:** 10.1021/acsbiomaterials.5c01884

**Published:** 2026-02-24

**Authors:** Patrizia Nadia Hanieh, Noemi Fiaschini, Anna Scotto d’Abusco, Alessia Mariano, Valeria Palumbo, Manuel Scimeca, Mariano Venanzi, Francesca Cavalieri, Carlo Abbate, Maurizio Mattei, Luigi Gentile, Antonio Rinaldi, Roberta Bernardini, Alberto Migliore

**Affiliations:** 1 Nanofaber S.r.l., Via Anguillarese 301, 00123 Rome, Italy; 2 Department of Biochemical Sciences Alessandro Rossi Fanelli, 9311 Sapienza University of Rome, Piazzale Aldo Moro 5, 00185 Rome, Italy; 3 Department of Experimental Medicine, 9318 University of Rome Tor Vergata, Via Montpellier 1, Rome 00133, Italy; 4 Department of Chemical Science and Technologies, 9318 University of Rome Tor Vergata, Via della Ricerca Scientifica 1, 00133 Rome, Italy; 5 Interdepartmental Center for Comparative Medicine, Alternative Techniques and Aquaculture (CIMETA), 9318 University of Rome Tor Vergata, Via Montpellier 1, Rome 00133, Italy; 6 Department of Chemistry, University of Bari and CSGI (Center for Colloid and Surface Science), via Orabona 4, 70125 Bari, Italy; 7 Department of Clinical Sciences and Translational Medicine, University of Rome Tor Vergata, Via Montpellier 1, Rome 00133, Italy; 8 San Pietro Fatebenefratelli Hospital, Via Cassia 600, 00189 Rome, Italy

**Keywords:** microsponges, rheumatoid arthritis, intra-articular delivery, sustained release, advanced therapies, pathological synovial fluid

## Abstract

Rheumatoid arthritis (RA) is a debilitating autoimmune disease characterized by chronic synovial inflammation and progressive joint destruction. Methotrexate (MTX) remains the gold standard in RA therapy, yet systemic administration often provides suboptimal joint targeting and causes dose-limiting toxicity. This study investigates intra-articular (IA) administration of an MTX-loaded Microsponge (MTX-MSP) as a localized strategy to enhance drug retention while minimizing systemic exposure. MTX-MSP is synthesized using hyaluronic acid-based cross-linking and MTX loading. Drug release and carrier mass loss were evaluated in a pathological human synovial fluid (HSF), and rheological and Fourier transform infrared spectroscopy analyses assess viscoelastic behavior and drug–carrier interactions. Biocompatibility and anti-inflammatory activity are tested in primary RA fibroblast-like synoviocytes, while therapeutic efficacy is evaluated in a collagen-induced arthritis rat model. MTX-MSP provides sustained release in HSF for 14 days, minimizes burst effects, and preserves structural integrity. Rheological profiling confirms injectability and interaction with the pathological synovial fluid, enhancing the elastic response. *In vitro*, MTX-MSP reduces IL-6, TNF-α, and IL-1β expression at gene and protein levels, outperforming free MTX. *In vivo*, weekly IA injections improve histological scores in treated and contralateral joints, suggesting systemic immunomodulation. MTX-MSP thus achieves prolonged release, anti-inflammatory efficacy, and reduced systemic toxicity, representing a promising IA formulation for RA management.

## Graphical abstract

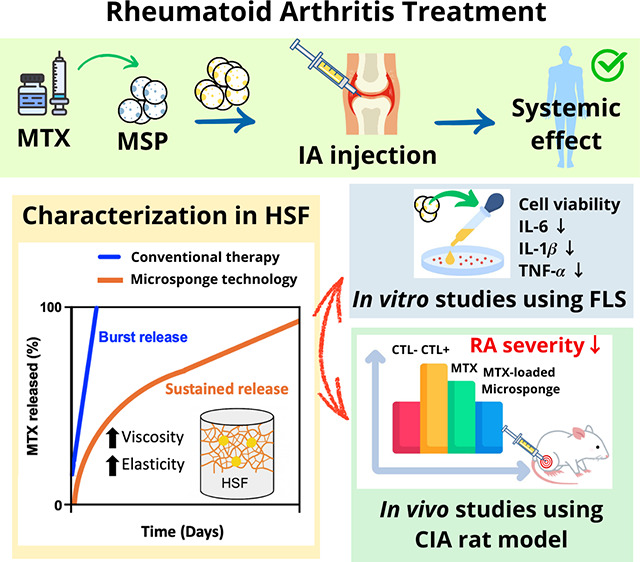

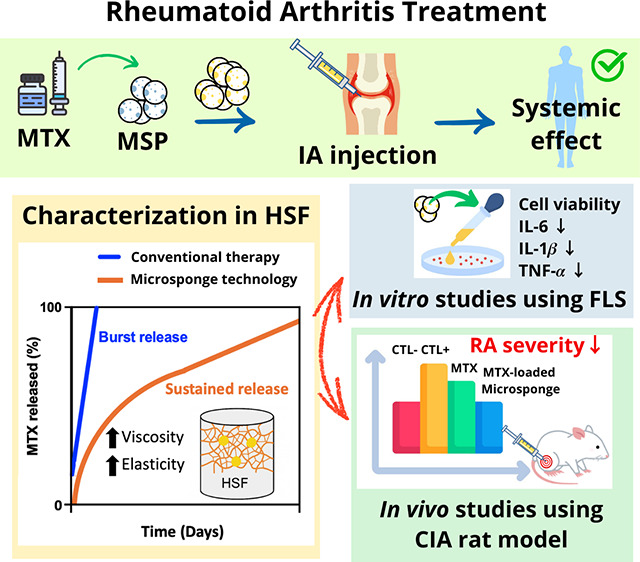

## Introduction

Rheumatoid arthritis (RA) is a systemic autoimmune disorder characterized by chronic synovial inflammation, pain, stiffness, and, ultimately, joint destruction.^1^ Affecting an estimated 17.6 million people worldwide, with particularly high prevalence in Europe and the United States, RA imposes a heavy burden on patients’ quality of life and functional capacity.^2^ The disease often leads to long-term disability, significantly impairing daily activities. Methotrexate (MTX) is widely recognized as the first-line treatment for RA due to its affordability and proven therapeutic benefits. It serves as a foundational agent in international treatment guidelines and is commonly combined with other disease-modifying antirheumatic drugs (DMARDs) to enhance efficacy.^3^

Although MTX remains a cornerstone in RA management by providing symptom relief and slowing disease progression, it rarely leads to complete remission. Notably, approximately 30–45% of RA patients experience adverse effects, including hepatotoxicity, gastrointestinal disturbances, renal insufficiency, and, in some cases, severe complications such as pancreatic cancer.^4^ Treatment in elderly patients is further complicated by comorbidities such as renal impairment or chronic lung disease, increasing susceptibility to side effects and often correlating with more aggressive disease progression.

Usually, MTX is administered once weekly, either orally or parenterally, at low doses ranging from 7.5 to 25 mg per week. Although oral administration is convenient, its efficacy is limited by gastrointestinal metabolism and the need for regular dosing, which elevates the risk of bleeding and fluid retention.^5^ Parenteral administration bypasses first-pass metabolism and improves bioavailability but can still result in off-target toxicity, variable systemic exposure, and suboptimal drug accumulation at inflamed joints.^6^

In this context, intra-articular (IA) injection emerges as a targeted delivery strategy, allowing direct deposition of the drug into the joint space to enhance local bioavailability and minimize systemic exposure.^7,8^ While IA delivery is widely used for corticosteroids, MTX has also been explored for this route, particularly in patients with oligoarticular or treatment-resistant RA and in juvenile idiopathic arthritis, aiming to control localized inflammation while limiting systemic toxicity.^9^ IA administration of MTX has demonstrated clinical benefits in terms of reducing synovial inflammation and improving joint function, with a low incidence of adverse effects.^10^

Nevertheless, the therapeutic effect of free MTX in the joint is often short-lived due to rapid clearance via lymphatic drainage and enzymatic degradation, necessitating repeated injections. Moreover, poor aqueous solubility and slow dissolution further hinder its retention and efficacy in the joint space.^11^

To overcome these limitations, a range of advanced drug delivery systems, such as nanoparticles, microparticles, liposomes, micelles, and hydrogels, have been designed to prolong IA residence time and modulate release kinetics.^12–14^ However, many formulations exhibit an initial burst release followed by a slow-release phase,^15^ and anti-RA agents often possess short intra-articular half-lives, further limiting sustained effectiveness.^16^

Considering the drawbacks of current strategies, enhancing the therapeutic profile of MTX while minimizing systemic toxicity remains a key goal. Optimizing its IA delivery through innovative formulations is a major focus of ongoing research in rheumatology and pharmaceutical science.

In our previous work,^17^ we demonstrated that the MTX-loaded Microsponge, porous spherical polymeric particles, enables efficient drug loading and sustained release with minimal initial burst over extended periods. Subcutaneous administration of this platform more than doubled therapeutic efficacy compared to conventional MTX dosing owing to prolonged systemic availability and a marked reduction in side effects.

Building on these results, the present preclinical study evaluated IA delivery of the MTX-loaded microsponge as a targeted strategy to increase the local drug concentration, limit systemic exposure, and overcome the rapid joint clearance that limits free MTX efficacy.

To this end, the physicochemical properties and release kinetics of MTX-loaded Microsponge formulation were evaluated in the presence of a pathological synovial fluid. In particular, *in vitro* studies using patient-derived samples confirmed that the MTX-loaded Microsponge enabled sustained drug release under inflammatory conditions. Rheological evaluations were performed to analyze interactions with a pathological synovial fluid and to confirm the formulation’s injectability and retention within the joint. Cytocompatibility and anti-inflammatory effects were investigated in primary synoviocytes isolated from RA patients.

Finally, a pilot *in vivo* study involving IA administration in a rat model of RA was conducted to assess the therapeutic efficacy of the formulation.

## Results and Discussion

In this work, the IA administration of the MTX-loaded Microsponge was evaluated, building on the strong foundation provided by our previous study,^17^ where subcutaneous administration demonstrated promising improvements in methotrexate bioavailability and therapeutic efficacy in the preclinical studies on rats affected by RA.

The shift to IA delivery aims to enhance therapeutic effects directly within affected joints while reducing systemic drug exposure and related side effects, concentrating treatment at the primary sites of inflammation.^18^

Supporting this approach, our earlier work^19^ demonstrated that the MSP platform, following IA injection, shows prolonged residence within the joint cavity, maintaining structural integrity and releasing its payload in a sustained manner. This extended retention is particularly advantageous under chronic inflammatory conditions such as RA, where continuous exposure to therapeutic agents at the site of inflammation is critical for effective disease control. Importantly, that study also provided robust evidence for the safety and biocompatibility of the MSP. Histological evaluations confirmed that IA injection did not provoke inflammatory responses or cause structural damage to joint tissues. The particles underwent gradual biodegradation without accumulation, further supporting their suitability for repeated or long-term use in joint-targeted therapies.

By integration of MTX into this previously validated delivery platform, the current study seeks to exploit the synergistic benefits of a disease-modifying antirheumatic agent with a localized, biocompatible carrier system. This strategy not only addresses the pharmacokinetic limitations of systemic MTX therapy but also supports sustained drug action at the pathological site. Taken together, the results from both studies reinforce the therapeutic potential of MSP for IA applications, offering a promising route for improving the management of chronic joint diseases.

An extensive physicochemical characterization of the Microsponge platform, both unloaded and methotrexate-loaded, was previously reported.^17^ SEM analysis showed spherical microparticles with a homogeneous open-porous architecture, while confocal imaging confirmed a nanoporous network and uniform drug distribution. Particle size analysis by laser diffraction under swollen conditions revealed mean diameters of ∼30 μm for empty MSP, increasing to ∼60 μm after methotrexate loading. All formulations displayed good colloidal stability and were endotoxin-free.

In the present work, characterization efforts are specifically directed toward evaluating the intra-articular performance of this system and clarifying its behavior in the presence of a pathological synovial fluid.

### Release of Methotrexate from the Microsponge and Carrier Mass Loss in the Synovial Fluid under Pathological Conditions

While a previous study^17^ focused on MTX release profiles in PBS to elucidate mechanistic aspects of the Microsponge system, the current evaluation in a human synovial fluid provides critical insights into the real-world performance of intra-articular therapies under physiopathological conditions.

The release profile of methotrexate over time in HSF, shown in Figure 1A, clearly highlights the difference in drug release kinetics between free MTX and MTX loaded in the Microsponge.

**Figure 1:**
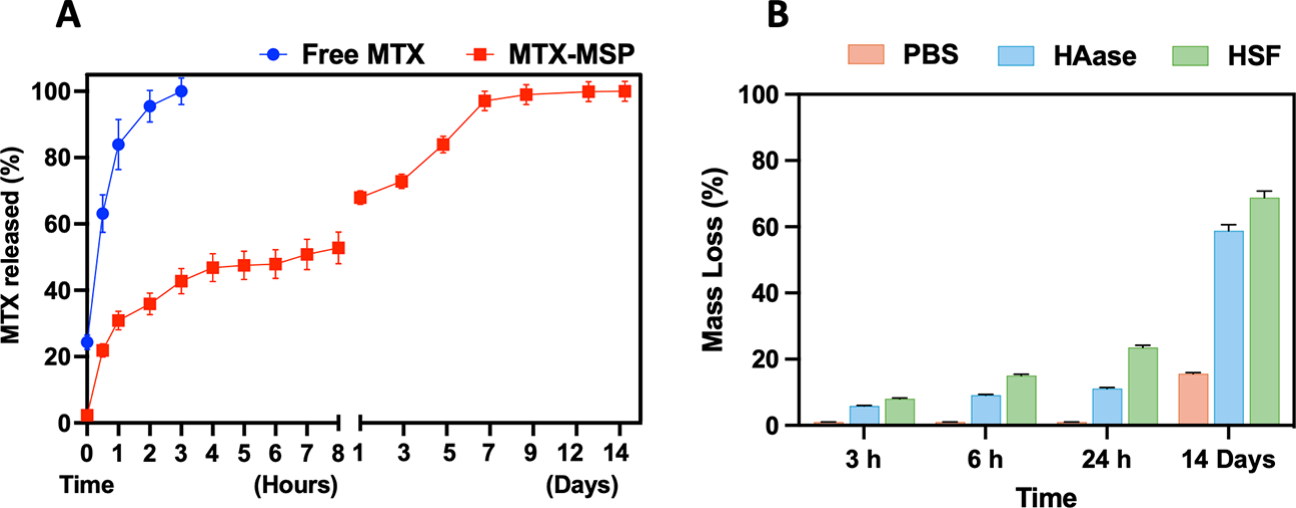
(A) Release profile of methotrexate using the free MTX formulation (blue line) and from the MTX-loaded microsponge (MTX-MSP, red line) over time in HSF. (B) *In vitro* degradation profile of MTX-MSP expressed as percentage mass loss over time in PBS, hyaluronidase (HAase), and HSF. The results are expressed as means ± standard deviation and were obtained from three independent experiments performed in triplicate.

At the initial time point (0 h), the commercial formulation (free MTX) already shows a release of approximately 24%, whereas no detectable release (∼2%) is observed for the MTX-MSP group, indicating a clear difference in immediate availability.

After 30 min, the free MTX rapidly releases nearly 63% of the drug, while the MTX-MSP system releases only 22%. This trend continues over time, with the free MTX reaching over 84% release within 1 h and reaching 100% at 3 h. Conversely, the MTX-MSP system demonstrated a markedly slower and sustained release, reaching only 31% at 1 h and 43% at 3 h. By 24 h, the MTX-MSP group had released 68%, confirming the extended-release kinetics of the microsponge-based formulation. The sustained release behavior was maintained over several days, with 97% of MTX released by day 7 and 100% achieved by day 14.

These data strongly suggest that the Microsponge system provides prolonged drug delivery, likely mediated by diffusion-regulated mechanisms within the porous polymer matrix. Such diffusion is facilitated by the physical entrapment of MTX within the hydrogel-like internal architecture, allowing gradual outward movement through water-filled channels.

In pathological joint environments such as those associated with RA, the complex composition of a human synovial fluid markedly influences drug release profiles. Elevated levels of high-molecular-weight hyaluronic acid and proteins, such as albumin, increase fluid viscosity and reinforce the microstructural network, thereby reducing molecular diffusion and prolonging drug retention. Furthermore, the presence of proteolytic enzymes, particularly matrix metalloproteinases, may contribute to the degradation of polymer-based delivery systems, potentially altering their performance depending on the susceptibility of the carriers to enzymatic cleavage. Recent evidence by Singh et al.^16^ highlights how enzymatic activity in arthritic joints can compromise the structural integrity of biodegradable systems, thus modulating drug release kinetics, in line with the trends observed in our system. This underlines the importance of designing systems capable of resisting or responding to such enzymatic stressors.

To assess whether the Microsponge formulation meets these requirements and to elucidate whether the observed MTX release profile is governed solely by diffusion or is also influenced by carrier degradation, the enzymatic stability of MTX-MSP was evaluated by gravimetric mass loss analysis (Figure 1B).

Within the early time points (3 and 6 h), MTX-MSP exhibited negligible mass loss in PBS, while slightly higher values were observed in the presence of HAase and HSF, indicating the onset of enzyme-mediated surface erosion (≤10–15%). At these short incubation times, degradation remained minimal and was clearly dependent on the presence of enzymatic activity.

By 24 h, MTX-MSP had no appreciable mass loss in PBS, confirming the intrinsic structural stability of the carrier under nonenzymatic conditions. In contrast, a moderate but clearly detectable mass loss was observed in the presence of hyaluronidase (∼11%) and human synovial fluid (∼23%), indicating that enzymatic activity contributes to progressive carrier erosion. The more pronounced mass loss detected in HSF compared to hyaluronidase alone can be attributed to the complex enzymatic composition of a pathological synovial fluid, which contains not only hyaluronidase but also multiple matrix metalloproteinases and inflammatory proteases acting synergistically on extracellular matrix components.^20^

Importantly, despite the detectable early mass loss in enzymatic and synovial-like conditions, the Microsponge retained substantial structural integrity within the first 24 h, a time window that is critical for early intra-articular drug retention. This behavior is consistent with previous studies on cross-linked or supramolecular HA-based systems, which typically undergo partial surface erosion rather than rapid bulk degradation when exposed to hyaluronidase or synovial enzymes, thereby preserving short-term stability.^21,22^

Upon prolonged incubation (14 days), the mass loss increased markedly under enzymatic and synovial-like conditions, reaching approximately 55–60% in hyaluronidase and ∼65–70% in HSF, whereas PBS-exposed samples showed measurable mass loss only at this late time point (∼15%), suggesting slow, nonenzymatic relaxation or dissolution of the HA-based network over extended incubation times. Comparable long-term degradation profiles have been reported for injectable HA-based hydrogels and collagen/HA composite biomaterials exposed to enzymatic environments, where delayed erosion supports sustained drug release while maintaining initial carrier integrity.^23^ Overall, these results support a release mechanism characterized by an initial diffusion-dominated phase under nonenzymatic conditions, followed by a degradation-assisted contribution that becomes significant only under enzymatic and synovial-like environments and at longer time points. Such a dual behavior has been widely recognized as advantageous for IA drug delivery systems, as it enables early retention of the carrier combined with controlled, inflammation-responsive degradation under pathological joint conditions.^21^

To better understand how methotrexate is incorporated into and retained within the MSP carrier, FTIR spectroscopy was performed. Detailed FTIR spectra and band assignments for empty MSP, free MTX, and MTX-loaded MSP are reported in the Supporting Information (Figure S1). The analysis confirmed that MTX is mainly incorporated through noncovalent interactions, such as hydrogen bonding and electrostatic forces, without significant alterations to the polymer matrix structure.^24,25^ These findings support the sustained release profile observed and the structural stability of the formulation prior to administration.

This mode of interaction is advantageous for environment-responsive drug delivery: in the absence of specific cleavage stimuli, the polymer matrix is expected to remain structurally stable, while in inflammatory, enzyme-rich settings, it may gradually undergo degradation, thereby facilitating drug liberation. Such a dual mechanism, diffusion under nonpathological conditions and enzymatic responsiveness under inflammatory ones, provides a plausible explanation for the extended release observed in the synovial fluid and supports the rational design of a joint-specific delivery platform tailored to the dynamic biochemical environment of inflammatory diseases.

### Rheological Assessment of the Microsponge in Arthritic Conditions

To complement the release studies and better understand the diffusion constraints within the biological environment, rheological characterization of all formulations in HSF was performed, as the viscosity and viscoelastic properties of the synovial fluid are known to significantly influence drug mobility and release kinetics from drug carriers. Therefore, comprehensive rheological characterization was conducted to investigate the mechanical behavior of the pathological synovial fluid and its interaction with the MSP, either unloaded or loaded with MTX. Rheological measurements were conducted at 37 °C to simulate the in situ postinjection behavior of the formulations within the joint cavity under physiological conditions. This temperature setting allows assessment of viscoelastic responses relevant to the intra-articular environment, rather than preinjection handling.

In parallel, rheological measurements were also conducted in PBS at 25 °C to assess the injectability and flow properties of the formulations prior to administration.

The flow behavior of the different systems is summarized in Figure 2, where panel (A) shows the apparent viscosity as a function of the shear rate and panel (B) presents the shear stress versus shear rate curves.

**Figure 2:**
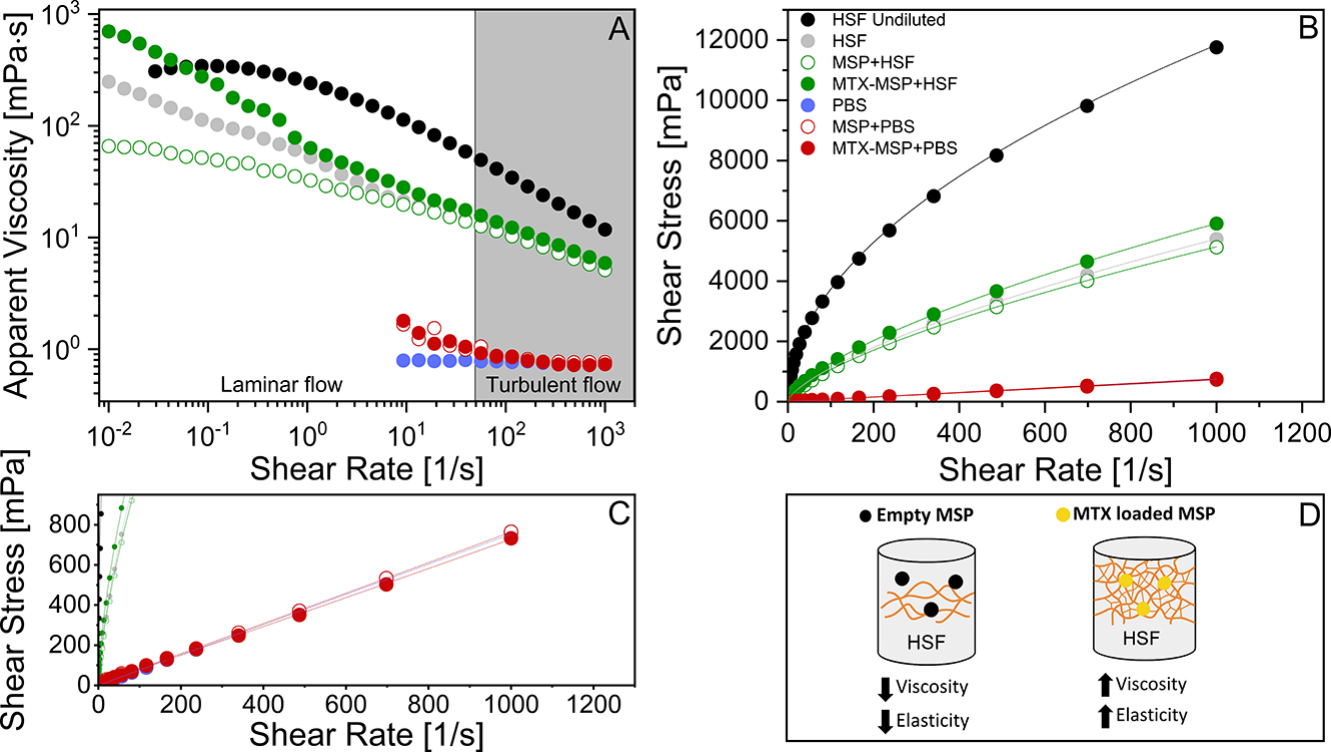
Flow curves of the samples showing (A) apparent viscosity as a function of the shear rate and (B) shear stress as a function of the shear rate. The continuous lines in panel (B) represent fits to the Herschel–Bulkley model (eq 2, Experimental Section) at 37 °C; (C) zoom of panel (B) showing that the samples PBS, MSP+PBS, and MTX-MSP+PBS are essentially identical; (D) schematic illustration of the interactions between both empty and loaded MSP and HSF.

The gray-shaded region in panel (A) marks the onset of turbulent flow, beyond which viscosity measurements become less reliable. The undiluted pathological synovial fluid from RA patients (HSF undiluted, black circles) exhibits classic pseudoplastic behavior, characterized by relatively high viscosity at low shear rates. Upon dilution with PBS (gray circles), the HSF overall viscosity decreases significantly, particularly in the low-shear regime. This reduction can be explained by ionic screening effects caused by PBS ions, which disrupt the electrostatic interactions among charged macromolecules such as hyaluronic acid and proteins, thereby weakening the microstructural network that normally resists deformation at a low shear rate. This observation aligns with other studies, demonstrating that increasing ionic strength through dilution with PBS significantly reduces the viscosity of HA solutions by weakening electrostatic interactions.^26,27^

When empty MSP is dispersed with HSF (open green circles), the pseudoplastic flow behavior is largely retained, although viscosity slightly decreases compared to HSF. The presence of MSP may contribute to slight alterations in the microstructural organization and molecular interactions within the fluid.^26^ A similar trend is observed for empty MSP dispersed in PBS (open red circles), where a mild yield–pseudoplastic profile emerges with generally lower viscosity.

The introduction of a methotrexate-loaded MSP markedly alters this behavior. Dispersions with MTX-loaded MSP in HSF (filled green circles) show a measurable yield stress and higher viscosity at low shear rates compared to both HSF undiluted and the empty MSP and HSF mixture, as reported in Table S1, Supporting Information. In particular, the increase in yield stress (σ_γ_) and consistency index (*K*) observed for MTX-loaded MSP relative to both empty MSP and HSF alone indicates the formation of a more cohesive microstructure, characterized by enhanced resistance to deformation under low-shear conditions. This behavior is consistent with additional physical interactions between MTX, the HA-based microsponge network, and high-molecular-weight synovial components, which reinforce the internal network structure of the dispersion.

This suggests that drug loading enhances the microstructural cohesion and network stability of the dispersion under low deformation, likely through interactions between MTX, the polymer matrix, and components of the synovial fluid (e.g., hyaluronic acid and proteins), thereby reinforcing the internal structure and further influencing diffusion-controlled release kinetics.

Similarly, MTX-loaded MSP in PBS (filled red circles) displays increased viscosity and yield stress relative to empty MSP dispersions, confirming the reinforcing effect of MTX incorporation.

In Figure S2, Supporting Information, our results confirm trends previously reported in the literature,^28^ showing a crossover between the storage modulus (*G*′) and the loss modulus (*G*″) at high frequencies, a sign of partial recovery of the fluid’s elastic properties.

According to the literature, the synovial fluid exhibits frequency-dependent viscoelasticity: it behaves more like a viscous fluid at low oscillation frequencies (e.g., slow joint movements) and is more elastic at higher frequencies (rapid movements). However, in RA, pathological synovial fluids become more viscous-dominated, with *G*′ falling below values observed in healthy fluids above ∼0.1 Hz.^29^

When HSF is diluted with PBS, a similar crossover pattern is observed, reflecting a mechanical weakening of the viscoelastic matrix.

Empty MSP consistently maintains a higher loss modulus (*G*″) even at elevated frequencies, reflecting a predominantly viscous response. On the other hand, MTX-loaded MSP shifts the crossover point toward lower moduli, indicating an improved elastic response, suggesting partial restoration of viscoelastic properties often lost in RA.^30^ Importantly, these rheological differences are observed within the shear rate and frequency ranges typically experienced during joint motion (≈0.01–100 s^–1^), supporting the relevance of the observed rheological reinforcement as a predictor of intra-articular retention.

### Injectability Assessment via Rheology of the Microsponge Formulation

Since injectability is a key requirement for IA administration, additional rheological tests were conducted at 25 °C of the formulations prior to injection, as shown in Figure 3.

**Figure 3:**
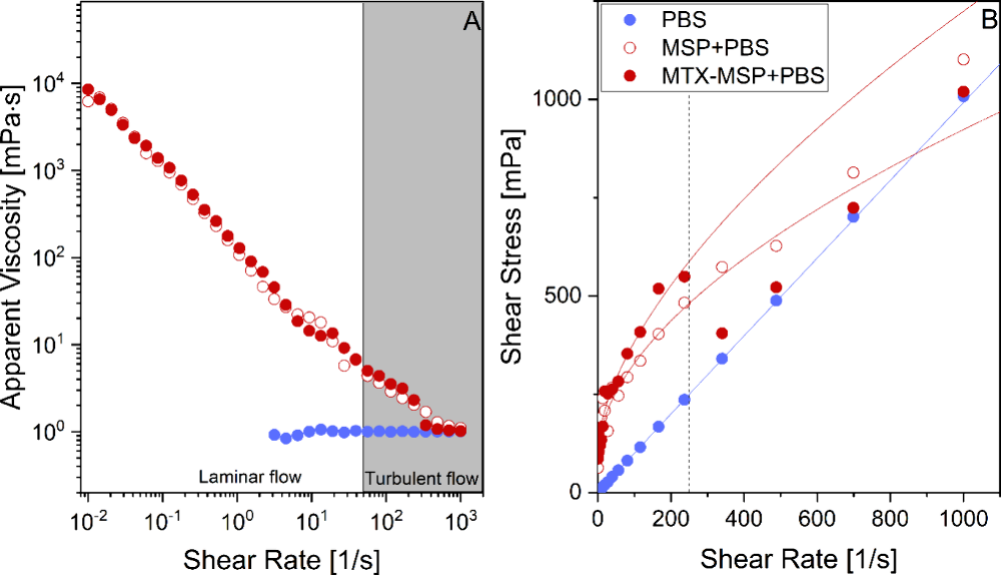
Flow curves of the samples showing (A) apparent viscosity as a function of the shear rate and (B) shear stress as a function of the shear rate. The continuous lines in panel (B) represent fits to the Herschel–Bulkley model (eq 2, Experimental Section) at 25 °C; the fitting is performed considering data until the dotted line.

The data represent the flow curves of the PBS, MSP+PBS, and MTX-MSP+PBS, allowing estimation of syringability and injectability. Syringability is closely related to injectability but emphasizes user experience and ease of injection, often evaluated through injection force, smoothness of flow, and lack of clogging. From a quantitative perspective, injectability and syringability are generally considered acceptable if the injection force is <30 N for clinical comfort, a smooth flow is observed with no sharp increases in resistance, and minimal plunger rebound or sticking occurs. An estimate of the injection force could be obtained through a generalized form of the Hagen–Poiseuille equation adapted for power-law fluids,^31^ as shown in eq S1, Supporting Information.

According to the flow parameters obtained, the injection force for all formulations remains well below 1 N, indicating outstanding syringability. Notably, the MTX-loaded MSP shows a higher viscosity and shear stress than the unloaded system yet still within acceptable ranges for clinical injection. This confirms the suitability of the formulation for IA delivery through standard needles without the requirement of excessive force.

### *In Vitro* Evaluation of the MTX-Loaded Microsponge on Human FLSs

To evaluate the advantages of administering MTX-loaded MSP compared with MTX alone, experiments on human primary FLSs were performed. First, to assess a potential cytotoxic effect, two different concentrations, 25 and 50 μg/mL of MTX alone or inside MSP, have been tested on cells, finding that cells were viable under all our experimental conditions (Figure 4). These two concentrations were chosen based on previously published results.^32^

**Figure 4:**
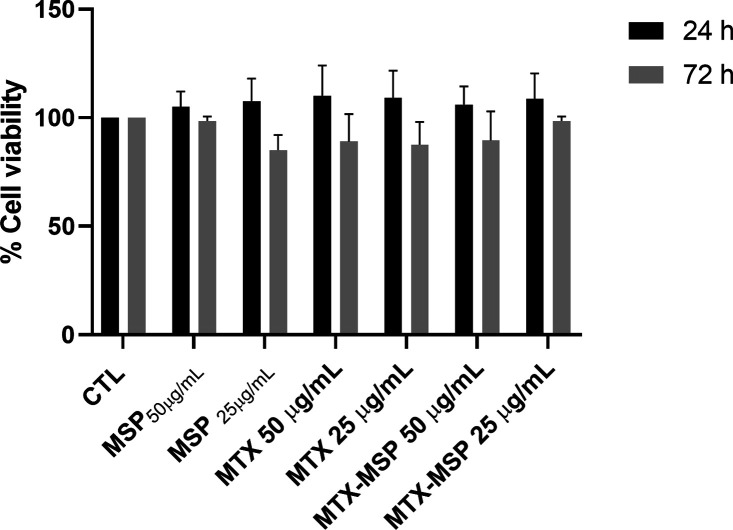
Cell viability was assessed by the Trypan Blue method, and FLSs were treated with two different concentrations of empty MSP, free MTX, and MTX-loaded MSP (50 and 25 μg/mL), for 24 and 72 h. The cell viability of treated samples was normalized to the untreated cells, which is reported as 100% (CTL).

Next, we evaluated the efficacy of MTX-loaded MSP to decrease the proinflammatory cytokine gene expression compared to both free MTX and empty MSP. MTX-loaded MSP was effective to decrease the IL-6 mRNA expression level at both 25 and 50 μg/mL compared to untreated and empty MSP treated cells after 72 h. MTX alone was able to decrease the IL-6 level, too, in particular at 50 μg/mL, even if to a lower extent with respect to MTX-loaded MSP (Figure 5). Regarding the IL-1β mRNA expression level, MTX alone was effective only at 50 μg/mL, while it was completely ineffective at 25 μg/mL. When MTX was loaded into MSP, it was effective at both 50 and 25 μg/mL, even if the latter was not significant (Figure 5). The very interesting result is regarding the TNF-α mRNA expression level, which was decreased only by 25 μg/mL MTX-loaded MSP (Figure 5). Empty MSP was not effective at any analyzed concentrations.

**Figure 5:**
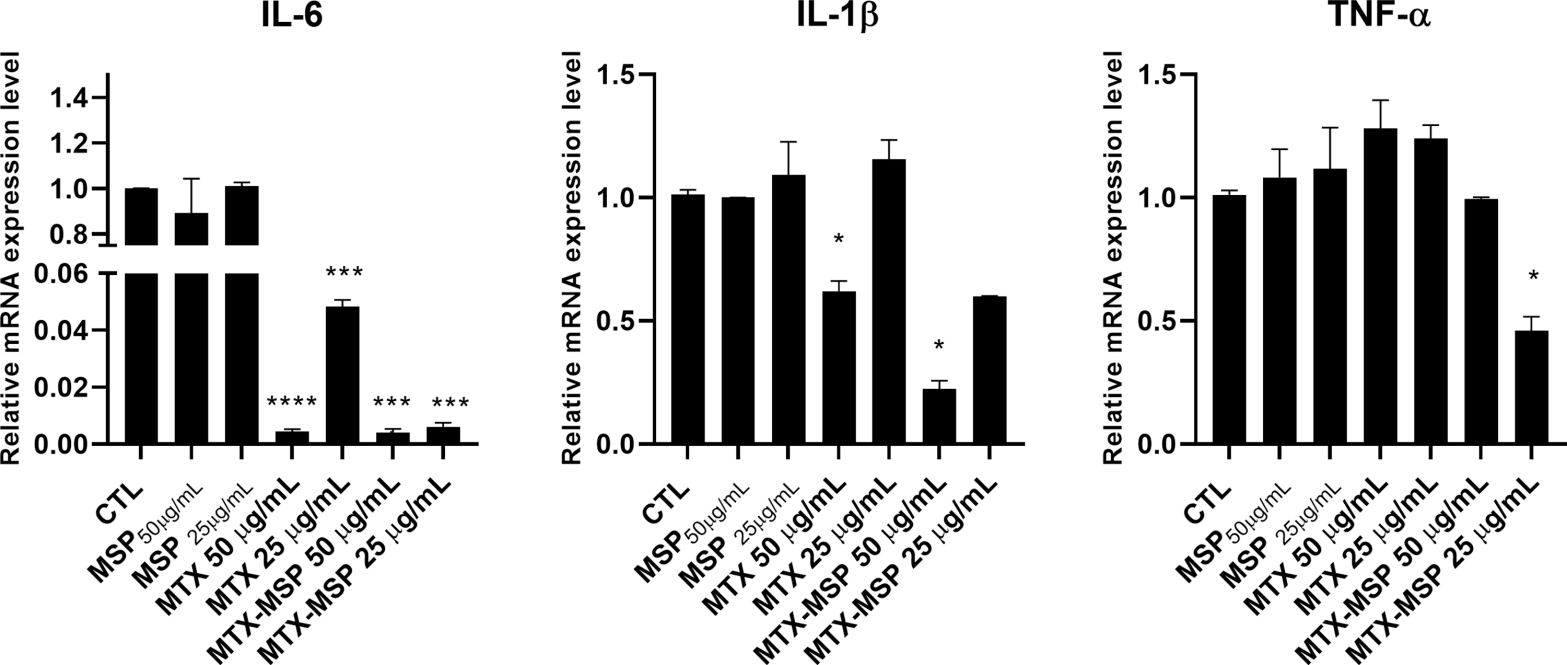
Effects of treatments on the cytokine mRNA expression levels. Cells were left untreated (CTL) or treated, for 72 h with 25 and 50 μg/mL MTX, MTX-loaded MSP containing 25 and 50 μg/mL MTX, and the corresponding MSP concentrations. After treatments, mRNA was extracted and analyzed by RT-PCR. IL-6, IL-1β, and TNF-α mRNA levels were reported as relative mRNA expression levels with respect to 18S mRNA (2^–ΔΔCt^ method). Results are expressed as means ± SEM of data obtained by three independent experiments. *p < 0.05; ***p < 0.005; ****p < 0.001 vs CTL.

Comparable results were obtained at the protein level by performing ELISA tests on cell culture media. Similarly, IL-6 protein was decreased by MTX-loaded MSP and MTX alone, even if to a lower extent (Figure 6). Regarding the IL-1β protein, it was statistically decreased by 50 μg/mL, both by MTX-loaded MSP and to a lower extent by MTX alone (Figure 6). Finally, only MTX-loaded MSP statistically decreased the level of TNF-α protein expression (Figure 6). Empty MSP was not effective at any analyzed concentrations. Similar results, even if less statistically significant, were obtained after 24 h of treatment both at mRNA and protein levels (data not shown).

**Figure 6:**
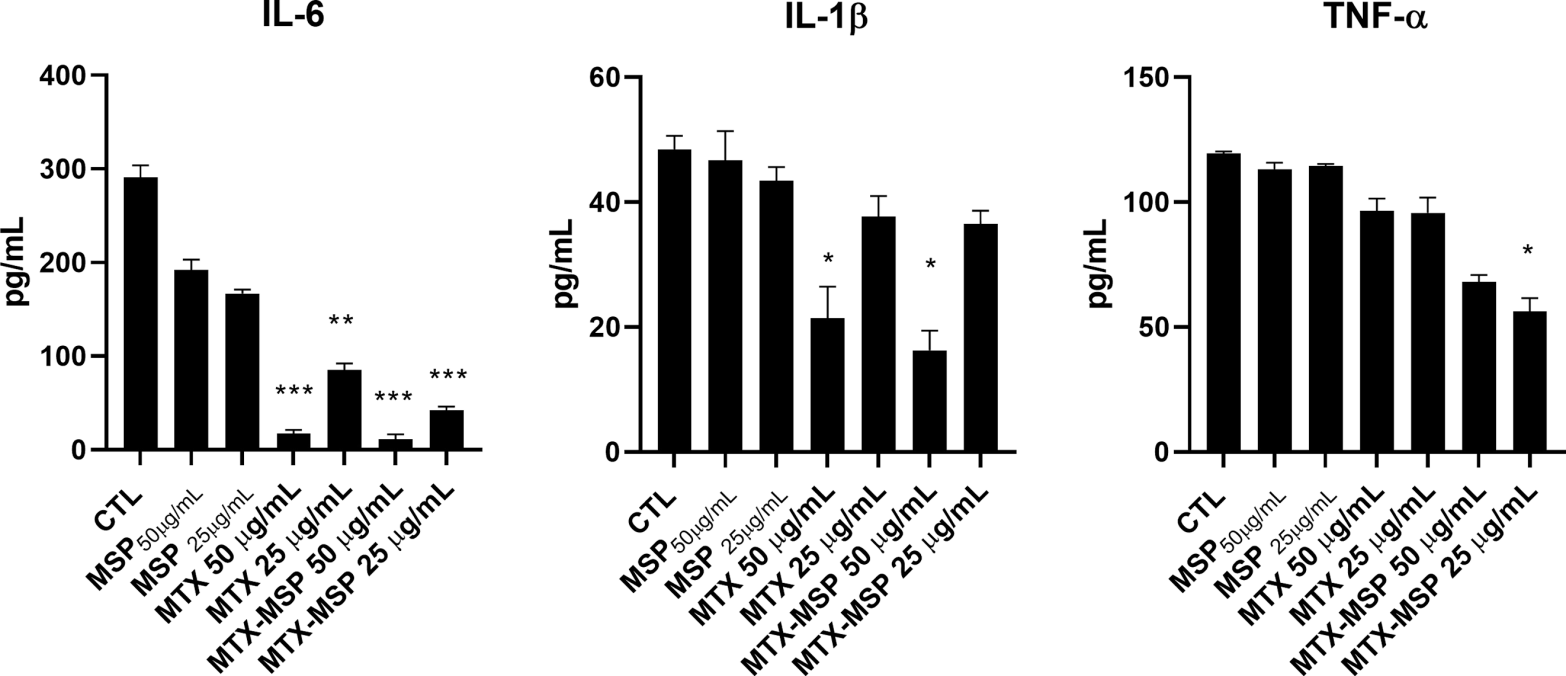
Effects of the treatments on cytokine protein levels. Cells were left untreated (CTL) or treated, for 72 h with 25 μg/mL and 50 μg/mL MTX, MTX-loaded MSP containing 25 μg/mL and 50 μg/mL MTX and the correspondent MSP concentrations. After treatments cell media were collected and analyzed by ELISA. IL-6, IL-1β and TNF-α protein levels were reported as pg/mL. Results are expressed as mean ± SEM of data obtained by three independent experiments. * *p* < 0.05; ** *p* < 0.01; *** *p* < 0.005 vs CTL.

### Joint Histopathology and Systemic Impact of IA Therapy with the Microsponge

Finally, histological analysis, derived from the pilot study conducted using the rat CIA model, was performed to evaluate the extent of joint damage and determine the protective effects of the different treatments. MTX-MSP centrifuged and MTX-MSP lyophilized formulations were included in the *in vivo* study to assess whether the lyophilization step affected the biological performance of the Microsponge following IA administration.

Hematoxylin and eosin (H&E) staining was applied to assess structural organization, cellular arrangement, matrix integrity, and tidemark preservation, providing a comprehensive evaluation of disease severity and therapeutic efficacy.

Figure 7, panel (a), provides representative H&E-stained joint sections that visually emphasize the structural alterations associated with disease progression and treatment response. In particular, panel (b) presents the colorimetric scoring matrix, where lighter shades of pink correspond to reduced histopathological severity, offering an immediate and intuitive visualization of treatment-associated improvements. These morphological aspects were also confirmed by the analysis of Masson-stained histological slides (Figure S4, Supporting Information).

**Figure 7:**
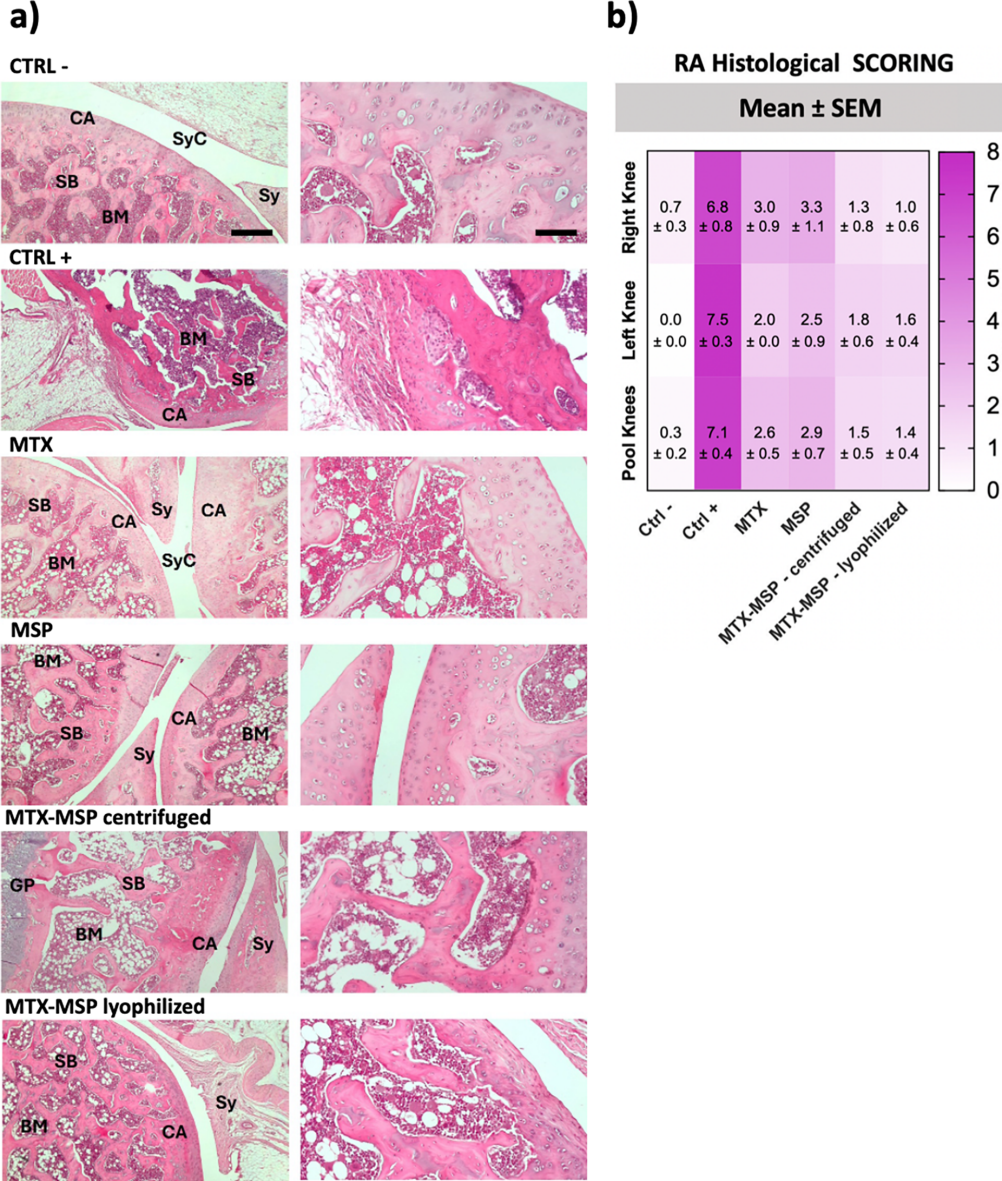
(a) Histological analysis of knee joints stained with hematoxylin and eosin. BM: bone marrow; BV: blood vessel; CA: cartilage; GP: growth plate; Me: meniscus; SB: subchondral bone; Sy: synovium; SyC: synovial cavity. Left column: magnification 5×, scale bar 500 μm. Right column: magnification 20×, scale bar 200 μm. (b) Histological RA scores for the right, left, and combined knees across different conditions. The scoring scale spans from 0 to 8, with darker shades representing higher values. Data are presented as the mean ± SEM.

In the Figure 8, panel (a), illustrates the administration schedule and its hypothesized systemic pathway, providing the framework for interpreting the subsequent histological findings.

**Figure 8:**
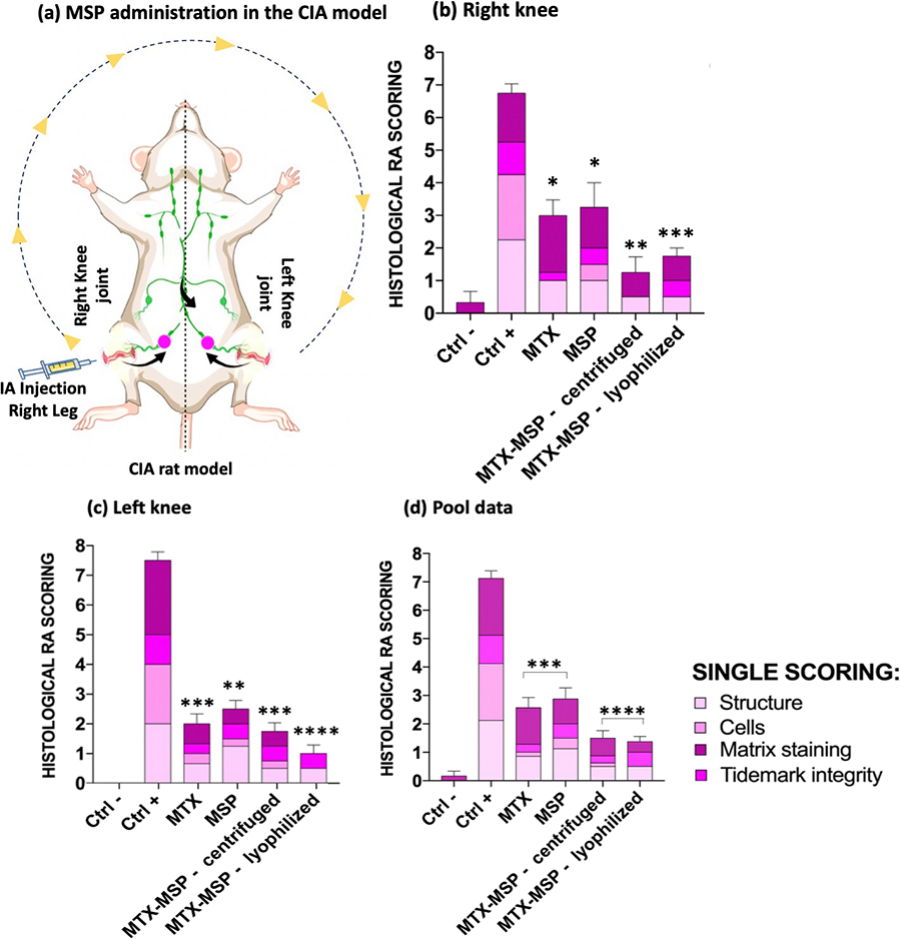
(a) Schematic of MSP injected intra-articularly in the CIA rat model and its hypothesized systemic pathway; (b) RA scores of rats in different groups for right analyzed knees; (c) RA scores of rats in different groups for left analyzed knees; (d) RA of rats in different groups for both analyzed knees. Data are presented as means ± SEM. *p < 0.05; **p < 0.01; ***p < 0.001; ****p < 0.0001 compared with the positive control.

Moreover, the qualitative and semiquantitative representation of H&E-stained sections is complemented by the numerical data, which quantitatively confirm and extend the histological observations. In particular, the final histological RA scores for each treatment group are comprehensively summarized in Figure 8, which presents separate scores for the right (panel (b)) and left limb (panel (c)) alongside the pooled data from both knees (panel (d)).

Within this context, H&E staining of both hind limbs in the negative control group revealed a well-preserved structural organization, an intact cellular arrangement, uniform matrix staining, and consistent tidemark integrity.

In contrast, as anticipated, the histological analysis of RA-afflicted rats in the positive control group demonstrated pronounced structural irregularities, disorganized cellular clustering, reduced matrix staining, and disrupted tidemark integrity. These pathological alterations were closely associated with an increased RA score in both limbs, further confirming the severity of joint damage (Figure 8, panel (d)). In both joints, MTX treatment resulted in a significant reduction of RA scores compared to the positive control, consistent with its established therapeutic effect.

MSP alone induced a partial improvement, while the combination of MTX and MSP achieved a greater reduction in pathological scores. As previously observed,^17^ a similar beneficial effect of MSP was also reported despite a different route of administration, an outcome that we hypothesized to be related to the high molecular weight of the hyaluronic acid used in the platform synthesis. This property, as reported in the literature, appears to play a crucial role in modulating inflammation by reducing proinflammatory cytokine expression and promoting anti-inflammatory mediators,^33^ as well as by preserving chondrocyte function and attenuating macrophage-driven inflammation in arthritic conditions.^34^

Notably, both centrifuged and lyophilized formulations of MSP combined with MTX yielded the lowest histological RA scores (Figure 8, panels (b–d)), underscoring their superior efficacy compared to MSP or MTX administered alone. While slight differences between centrifuged and lyophilized preparations could be observed, the overall trend clearly indicates that the coformulation with MTX provided the most consistent and significant protection against joint damage.

In the negative control (CTRL−), the cartilage and subchondral bone were preserved, marrow cavities appeared intact, and the synovial lining was thin and regular, reflecting a normal joint histoarchitecture.

In sharp contrast, the positive control (CTRL+) showed severe pathology, including cartilage erosion, thickened and hyperplastic synovium, inflammatory cell infiltration, and disrupted subchondral bone structure features that directly correspond to the highest RA scores. Treatment with MTX or MSP alone partially ameliorated these pathological changes, resulting in reduced synovial proliferation and improved cartilage preservation, although residual irregularities persisted. Strikingly, the MTX-MSP combination preserved cartilage integrity, maintained subchondral bone organization, and markedly reduced inflammatory infiltration, in close agreement with the lowest histological scores observed in Figure 8.

According to the obtained results, the IA administration of a methotrexate-loaded Microsponge provides a highly targeted strategy for addressing the localized inflammation and damage characteristics of RA joints. Remarkably, in our study, the injection of the treatment solely into the right knee resulted in significantly positive outcomes not only in the treated knee but also in the contralateral (left) knee. This suggests that although the administration was confined to a specific joint, the therapeutic effects extended systemically. Such findings imply that the treatment likely triggered broader immunomodulatory mechanisms or led to the circulation of bioactive compounds, which facilitated a beneficial response in the untreated joint.

To further explore this aspect, systemic immunological analyses were performed, including ELISA assays for anticollagen type II antibodies and for the proinflammatory cytokines IL-1β, IL-6, and TNF-α. While anticollagen II antibodies and IL-6 levels did not show significant differences among experimental groups, IL-1β was significantly reduced in all treated groups compared to the positive control, with the strongest effect observed for both MTX-MSP formulations (centrifuged and lyophilized); instead, TNF-α levels were significantly decreased only in the MTX-MSP centrifuged group (detailed data are provided in Figure S3, Supporting Information).

These results indicate a selective and partial systemic modulation of inflammatory mediators, which alone does not fully explain the pronounced histopathological improvement, thereby supporting the contribution of local or tissue-specific mechanisms.

This systemic effect underscores the potential of intra-articular drug delivery systems to provide widespread therapeutic benefits beyond the targeted site of administration.

Taken together, the integrated evidence from Figures 7 and 8 provides a coherent and complementary view of the therapeutic efficacy. While the scoring system captures statistically significant reductions in disease severity across treatment groups, the histological sections vividly illustrate the structural preservation achieved by MSP when combined with MTX.

This dual approach reinforces the conclusion that MSP exerts a robust protective effect against joint damage in experimental RA, effectively preserving both cartilage and bone integrity, while intra-articular delivery ensures both local and systemic therapeutic benefits.

Importantly, these systemic effects are consistent with our previous preclinical study,^17^ in which MSP administered subcutaneously also produced significant histological improvements. Overall, these findings demonstrate that MSP, when combined with MTX, provides a consistent and reproducible therapeutic benefit across different routes of administration, highlighting its strong potential as an effective formulation for preserving joint integrity and mitigating disease progression in RA.

## Conclusions

This study establishes that intra-articular administration of the methotrexate-loaded Microsponge provides an effective therapeutic approach for RA, particularly in patients with oligoarticular or treatment-resistant RA and in juvenile idiopathic arthritis. The formulation enables sustained drug release directly within the pathological synovial fluid, minimizing the initial burst effect and preserving the carrier integrity over time. Taken together, release and degradation data indicate that MTX-MSP exhibits a diffusion-dominated release at early time points, supported by preserved carrier integrity, followed by a progressive, enzyme-responsive erosion under synovial-like conditions that contributes to sustained drug liberation over longer periods. Rheological studies revealed how MTX-MSP modulates and adapts to the viscoelastic properties of the pathological synovial fluid after injection, while syringeability tests confirmed favorable flow behavior and ease of administration through clinical-grade syringes.

Overall, these results demonstrate the potential of MTX-MSP as a long-acting intra-articular therapy, capable of maintaining therapeutic concentrations with fewer injections and lower systemic exposure. Such controlled delivery is particularly valuable in chronic inflammatory diseases such as RA, where continuous local drug availability is essential to suppress inflammation and protect joint integrity.

*In vitro* studies in primary human fibroblast-like synoviocytes showed that MTX-MSP significantly reduced the expression and secretion of key proinflammatory cytokines (IL-6, IL-1β, and TNF-α), outperforming free MTX. Importantly, *in vivo* results in a collagen-induced arthritis rat model revealed not only local therapeutic efficacy in the treated joint but also beneficial effects in the contralateral, untreated joint, indicating systemic immunomodulation despite localized administration.

Collectively, these findings identify MTX-MSP as a translationally relevant biomaterial platform that enhances the therapeutic profile of methotrexate, offering a promising long-acting strategy for the sustained management of chronic joint inflammation.

## Experimental Section

### Materials

Di­(imidazol-1-yl)­methanone (carbonyl diimidazole, CDI), 2,2′-diaminodiethyl disulfide dihydrochloride (Cys), dipotassium trisodium dihydrogen phosphate-hydrogen phosphate-dichloride (phosphate-buffered saline, PBS), and hyaluronidase (type IV, bovine testes) were purchased from Merck Italia (Milan, Italy). Sodium hyaluronate (HA, molecular weight of 1590 kDa) was obtained from Lifecore Biomedical (Chaska, MN, USA). Commercial methotrexate (Reumaflex prefilled syringe, 50 mg/mL) was provided by Alfasigma S.p.A. (Bologna, Italy). All other reagents and chemicals used were of analytical grade. The synovial fluid (HSF) was obtained from arthritis patients, who underwent arthroplasty surgery, at Santa Scolastica Hospital, Cassino, ASL Roma 2, and stored at −80 °C until further use.

### Drug Release in the Presence of the Synovial Fluid of an Arthritis Patient

Empty and MTX-loaded MSP were synthesized and characterized as previously described.^17^

Briefly, MSP was obtained through a one-pot self-precipitation and cross-linking process by adding hyaluronic acid to the cross-linker. Subsequently, methotrexate was loaded onto the MSP porous network, and unbound drug was removed by centrifugation. The resulting formulation was either used as the centrifuged suspension (MTX-MSP centrifuged) or freeze-dried and reconstituted prior to use (MTX-MSP lyophilized). Purification was performed under sterile conditions, and the endotoxin-free status was confirmed by limulus amebocyte lysate (LAL) testing.

While the previous formulation was developed for subcutaneous administration, the present protocol was adapted to simulate intra-articular delivery and to assess drug release behavior in a pathological synovial environment. MTX release from MSP was evaluated using the dialysis bag technique under conditions mimicking the inflammatory synovial fluid characteristic of RA. Specifically, 1 mg of MTX-loaded MSP was suspended in 0.5 mL of a 1:1 (v/v) mixture of PBS and HSF^16,35^ and then sealed within a preconditioned dialysis membrane (molecular weight cutoff 12–14 kDa; SpectraPore, Spectrum Laboratories, Inc., Rancho Dominguez, CA, USA). This MWCO range was selected to allow diffusion of free MTX (454 Da) while retaining high-molecular-weight synovial components, such as hyaluronic acid and albumin, inside the dialysis bag. This configuration preserves the pathological macromolecular environment of the synovial fluid and enables a direct comparison between free MTX diffusion and MSP-mediated release under RA-like conditions.

As RA causes a significant increase in the synovial fluid volume due to inflammation, the *in vitro* model volume was adjusted accordingly. While preclinical studies often assume a synovial fluid volume of around 50 μL per healthy rat knee,^36^ the model volume was increased to 500 μL to approximate the enlarged joint environment under arthritic conditions. This adjustment is also in line with clinical observations in human RA joints, where synovial fluid accumulation may reach several milliliters,^37,38^ thereby providing a more relevant setting for evaluating MTX release. The dialysis bag was immersed in PBS (external release medium) and maintained at 37 ± 1 °C under constant agitation at 200 rpm. At predefined time points, hourly during the first 8 h and then weekly for up to 14 days, aliquots of the external medium were collected and replaced with fresh PBS to maintain sink conditions. MTX concentrations in the release medium were quantified by UV–visible spectroscopy at 302 nm.

In parallel, to assess any potential influence of HSF on the drug itself, the experiment was also conducted using free MTX under identical conditions. All experiments were independently conducted in triplicate.

Moreover, Fourier transform infrared (FTIR) spectroscopy was performed to analyze drug–carrier interactions. Detailed experimental procedures are reported in the Supporting Information.

### *In Vitro* Enzymatic Degradation Studies

In order to elucidate whether MTX release from the Microsponge system is governed primarily by passive diffusion or is also influenced by the structural persistence and enzymatic stability of the carrier in synovial-like environments, *in vitro* degradation studies were performed.

These studies were carried out on the MTX-loaded microsponge by gravimetric mass loss analysis in the following media: (i) PBS, used as a nonenzymatic control to evaluate the intrinsic structural stability of the carrier; (ii) hyaluronidase (HAase, 10 U/mL^39^), used to selectively probe the enzymatic susceptibility of the HA-based MSP matrix; (iii) HSF, to reproduce a pathological synovial-like environment while preserving the complex enzymatic and proteolytic milieu characteristic of arthritic joints. The HSF dilution was selected to maintain consistency with the *in vitro* release experiments.

The initial dry mass of the samples was recorded (2 mg) prior to incubation. Samples were then incubated in 0.5 mL of each medium at 37 °C under continuous orbital agitation (100 rpm). At predetermined time points (3, 6, and 24 h and 14 days), samples were recovered by centrifugation, the supernatant was removed, and the remaining material was freeze-dried for 24 h. The dry mass was recorded before (*W*_0_) and after incubation (*W_t_*). The percentage mass loss was calculated according to the following equation:^23^

$$\mathrm{mass}\;\mathrm{loss}\left(\%\right)=\frac{W_{0}-W_{t}}{W_{0}}\times 100$$ 1

### Rheological Properties of Formulations in the Presence of the RA Human Synovial Fluid

Since the suitability of a formulation for IA administration depends not only on injectability but also on its ability to perform within the viscoelastic environment of the joint, rheological studies were carried out both in PBS at 25 °C, to assess preinjection flow behavior, and in the pathological synovial fluid at 37 °C, to evaluate the mechanical interactions influencing drug diffusion and release. This comprehensive analysis allowed correlation of the formulation’s rheological properties with its sustained release profile under biologically relevant conditions.

Rheological measurements were carried out to assess the viscoelastic and flow behavior of both unloaded and loaded MSP dispersed at a 1:1 (v/v) ratio in either PBS or HSF. HSF was collected from patients with RA to replicate the inflamed joint environment, and PBS served as the physiological control.

All measurements were performed using an Anton Paar MCR 302 Evolution Rheometer (Anton Paar, Austria) equipped with a cone–plate geometry (cone diameter: 49.96 mm; cone angle: 1°). The truncation gap between the cone and the lower plate was 106 μm (sample gap). Approximately 1 mL of each formulation was loaded onto a measurement plate. For tests in HSF, the temperature was maintained at 37 °C by using the integrated Peltier system to simulate physiological conditions.

Two types of rheological tests were conducted to characterize the formulations: stationary measurements and oscillatory measurements.

In the stationary measurements, the flow behavior was evaluated by recording viscosity and shear stress as functions of the shear rate, typically spanning from low to high values to capture both Newtonian and non-Newtonian characteristics. The data were fitted to the Herschel–Bulkley model,^40^ defined by the following equation:

$$\sigma \left(\mathrm{\gamma ̇}\right)=\sigma_{y}+K\left(T\right){\mathrm{\gamma ̇}}^{n}$$ 2

where σ is the shear stress (Pa), σ_*y*_ is the yield stress (Pa), *K*(*T*) is the consistency index (Pa s^*n*^), γ̇ is the shear rate (s^–1^), and *n* is the flow behavior index (dimensionless).

The Herschel–Bulkley model was selected because the pathological synovial fluid and all HSF-based dispersions exhibited yield–pseudoplastic behavior, characterized by a finite yield stress and shear-thinning flow. Full Herschel–Bulkley fitting parameters for all formulations at 25 and 37 °C are reported in Table S1 (Supporting Information).

When σ_*y*_ = 0, the Herschel–Bulkley model simplifies to the Ostwald–de Waele (power-law) model. The Herschel–Bulkley equation accounts for yield stress and describes both shear-thinning (pseudoplastic, *n* < 1) and shear-thickening (dilatant, *n* > 1) behaviors. Measurements were acquired in the shear rate range of 10^–2^–10^3^ s^–1^, maintaining laminar flow conditions up to 50 s^–1^.

In the oscillatory measurements, samples exhibiting pseudoplastic or yield–pseudoplastic behavior in flow measurements were further characterized in the linear viscoelastic regime (LVR) by using oscillatory shear tests. A frequency sweep was performed over a range of angular frequencies (0.1–200 rad/s) at a fixed strain within the LVR, previously identified via strain sweep tests. These measurements enabled the extraction of the storage modulus (*G*′) and loss modulus (*G*″), representing the elastic and viscous responses of the material, respectively.

### *In Vitro* Studies Using Human Primary Synoviocytes and Cell Treatments

Human primary fibroblast-like synoviocytes (FLSs) were isolated from synovial membranes,^41^ from patients with RA who underwent total knee arthroplasty.

The Research Ethics Committee, Sapienza University of Roma (#290/07, 29 March 2007), and ASL Lazio 2 (#005605/2019, 3 March 2019) approved the study, and full ethical consent was obtained from all donors. In brief, the synovial membrane was minced and treated with 1 mg/mL collagenase type IV and 0.25% trypsin for 2 h at 37 °C in agitation. Then, FLSs were cultured at 37 °C and 5% CO_2_ in DMEM (Merck Life Science, Darmstadt, DE) supplemented with l-glutamine, penicillin/streptomycin (Merck Life Science), and 10% fetal bovine serum (FBS) and grown to 80% confluence. All experiments were carried out with synoviocytes at first passage (p1), isolated from at least 3 different donors. Cells were left untreated (CTL) or treated for 24 and 72 h with 25 and 50 μg/mL MTX, MTX-loaded MSP containing 25 and 50 μg/mL MTX, and the corresponding MSP concentrations.

### Cell Viability

To assess a potential cytotoxic effect of different concentrations of MSP containing and not containing MTX and with MTX alone, the Trypan Blue method (Merck Life Science) was performed. Briefly, 80 × 10^3^ cells were seeded in a 24-well plate and left untreated (CTL) or treated with MSP and MSP + MTX and MTX alone, for 24 and 72 h.

### RNA Extraction and RT-PCR

Total RNA was extracted from untreated and treated FLSs, with a Blood/Tissues Total RNA extraction kit (Fisher Molecular Biology, Trevose, PA, USA), and the reverse transcription was performed according to the manufacturers’ instructions by OneScript Hot Reverse Transcriptase (Applied Biological Materials, Inc., Richmond, Canada).

Quantitative real-time PCR analysis was performed using an ABI Prism 7300 (Applied Biosystems, Thermo Fisher Scientific, Waltham, MA, USA). Amplification was carried out using a SensimixPlus SYBR Master mix (Bioline, London, UK). Primers (Table 1) synthesized by Biofab Research (Rome, Italy) were designed using Primer Express software (Applied Biosystems). Data were analyzed by the 2^–ΔΔCt^ method, determining the transcript abundance relative to the 18S housekeeping gene.

**Table 1:** RT-PCR Primer Sequences

gene accession number	primer forward primer reverse
IL-6	5′-GATGGATGCTTCCAATCTG-3′
NM_000600	5′-CTCTAGGTATACCTCAAACTCC-3′
TNF-α	5′-AGCTGAGAGATAACCAGCTGG-3′
NM_000594	5′-AGGACCTGGGAGTAGATGAGG-3′
IL-1β	5′-ACAGAATCTCCGACCACCACTA-3′
NM_000576	5′-TCCATGGCCACAACAACTGA-3′
18S	5′-CGCCGCTAGAGGTGAAATTC-3′
NM_003286	5′-CATTCTTGGCAAATGCTTTCG-3′

### ELISA

The amounts of IL-6, TNF-α, and IL-1β in the cell supernatant were determined using an enzyme-linked immunosorbent assay kit (Fine Test ELISA, Fine Biotech Co., Ltd., Wuhan, China) according to the manufacturer’s instructions. Optical density (O.D.) absorbance was measured at 450 nm by a microplate reader (NB-12-0035, NeBiotech, Holden, MA, USA).

### Ethical Approval in *In Vivo* Studies

All experiments were carried out at the Interdepartmental Center for Comparative Medicine, Alternative Methods, and Aquaculture at the University of Rome Tor Vergata. The animal studies received ethical approval from the Animal-Welfare Body (OPBA) and were authorized by the Ministry of Health under Legislative Decree no. 26/2014, in compliance with European Directive 2010/63/EU (Authorization No. 763/2021-PR, issued on October 12, 2021). This study was conducted and documented following ARRIVE (Animal Research: Reporting of *In Vivo* Experiments) guidelines (https://arriveguidelines.org/, accessed on 10/01/2022).

### Animal Husbandry

This study involved 23 male Wistar rats (Envigo, Rome, Italy) aged between 9 and 11 weeks. Before the experiment, the animals underwent a 7-day acclimatization period and were housed under standardized conditions: a 12 h light/dark cycle, a room temperature of 20 ± 2 °C, and 55% relative humidity. They had unrestricted access to a standard laboratory diet and tap water. A veterinary surgeon, responsible for laboratory animal welfare, was present throughout the study, and trained personnel oversaw animal care. Rats were assigned to experimental groups based on calculations performed using G Power Analysis.

### Animal Treatment for a Preclinical Study

The rats were randomly assigned six groups. To induce RA, five groups received an intradermal immunization of 200 μg of type II collagen from the bovine tracheal cartilage (Sigma C1188) dissolved in 0.05 M acetic acid/100 μL per rat, emulsified with an equal volume of incomplete Freund’s adjuvant (IFA) (Chondrex, Redmond, WA, USA) on day 7. A booster injection of 100 μg of collagen-IFA suspension was administered on day 15 following the same procedure (Figure 9).

**Figure 9:**
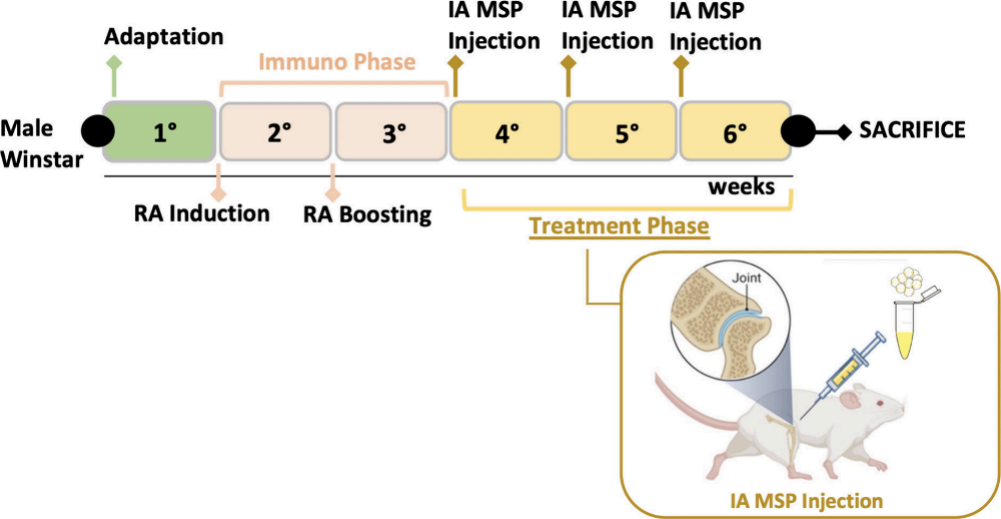
Experimental design of the preclinical study.

Following this, the animals were randomized into the following experimental groups:

1. Healthy nonarthritic as the negative control (3 rats);
2. arthritic untreated as the positive control (4 rats).

From day 22 to 42, immunized groups were subcutaneously administered with a single intra-articular injection every week of the following:

1. MTX 0.125 mg/rat in 0.08 mL of NS for the MTX group (4 rats);
2. MSP 1 mg/rat in 0.08 mL of NS for the MSP group (4 rats);
3. MTX-MSP centrifuged group: arthritic rats treated with 1 mg of MTX-MSP centrifuged/rat in 0.08 mL of NS (4 rats);
4. MTX-MPS lyophilized group: arthritic rats treated with 1 mg of MTX-MSP lyophilized/rat in 0.08 mL of NS (4 rats).

On day 43, all animals were anesthetized with ketamine (70 mg/kg) and metomidine hydrochloride (0.1 mg/kg). Euthanasia was performed via intracardiac blood sampling, followed by aortic arch excision. Posterior knee joints were collected for further analysis.

### Histological Analysis

Right and left hind limbs were fixed in formalin and subsequently decalcified using 0.5 M EDTA and 0.5% paraformaldehyde for 24 h. Decalcified specimens underwent dehydration through a graded alcohol series, followed by clearing with xylene and embedding in paraffin. Sections of the talocrural joint, each 5 μm thick, were stained with both H&E and Masson staining and placed on glass slides for histological examination under a light microscope. The joint’s histopathological alterations were assessed based on morphological characteristics including the structure, cells, matrix, and tidemark, as previously described.^42^ A cumulative scoring system ranging from 0 to 11 points was applied with higher scores reflecting increased tissue damage. The specific criteria for determining the histopathological score are detailed in a previous work.^17^

### Statistical Analysis

All quantitative data were obtained from a minimum of three independent experiments, each performed in duplicate or triplicate to ensure reproducibility and reliability. Results are expressed as mean values ± standard error of the mean (SEM), unless otherwise specified. Statistical analyses were conducted using two-way repeated measures analysis of variance (ANOVA) to evaluate differences over time and between treatment groups. When appropriate, Bonferroni’s post hoc test was applied to correct for multiple comparisons and identify pairwise differences. This statistical approach was chosen to account for the correlated nature of repeated measures and to enhance the precision of intergroup comparisons. All analyses were performed using GraphPad Prism software, version 5.0 (GraphPad Software, San Diego, CA, USA). A *p*-value <0.05 was considered indicative of statistical significance throughout the study. This approach ensured both statistical rigor and appropriate interpretation of longitudinal experimental data, particularly in evaluating treatment effects across time points and conditions.

## Supplementary Material



## Notes

FTIR spectra of the microsponge, free methotrexate, and methotrexate-loaded microsponge (Figure S1); Herschel–Bulkley fitting parameters of rheological measurements for all formulations in PBS and synovial-based media at 25 and 37 °C (Table S1); frequency sweep rheological experiments of all samples in the presence of a synovial fluid (Figure S2); estimation of the injection force using the Hagen–Poiseuille equation adapted for power-law fluids (eq S1); ELISA quantification of serum IL-1β and TNF-α levels at the end of the *in vivo* study (Figure S3); histological analysis of knee joints stained with Masson’s trichrome (Figure S4) (PDF)

## Abbreviations

RA: rheumatoid arthritis
MTX: methotrexate
IA: intra-articular
MTX-MSP: MTX-loaded microsponge
HSF: human synovial fluid
HAase: hyaluronidase
CIA: collagen-induced arthritis
H&E: hematoxylin and eosin staining
BM: bone marrow
BV: blood vessel
CA: cartilage
GP: growth plate
Me: meniscus
SB: subchondral bone
Sy: synovium
SyC: synovial cavity
CDI: carbonyl diimidazole
Cys: 2,2′-diaminodiethyl disulfide dihydrochloride
PBS: phosphate-buffered saline
HA: sodium hyaluronate
FLSs: human primary fibroblast-like synoviocytes
