# Supplementary material for: Intra-articular Methotrexate-Loaded Microsponge as an Adjuvant Strategy for Rheumatoid Arthritis: Localized Treatment with a Systemic Impact
Source: ACS Biomater Sci Eng. 2026 Feb 24;12(3):1603–15. doi: 10.1021/acsbiomaterials.5c01884 (PMC12976990; doi:10.1021/acsbiomaterials.5c01884)
Supplement: Supplementary file 1 [file ab5c01884_si_001.pdf]

Supporting Information

Intra-articular methotrexate-loaded Microsponge as an adjuvant strategy for rheumatoid arthritis: localized treatment with systemic Impact

Patrizia Nadia Hanieh[1,‡], Noemi Fiaschini[1,‡], Anna Scotto d'Abusco[2], Alessia Mariano[2], Valeria Palumbo[3], Manuel Scimeca[3], Mariano Venanzi[4], Francesca Cavalieri[4], Carlo Abbate[5], Maurizio Mattei[5], Luigi Gentile[6], Antonio Rinaldi[1,], Roberta Bernardini[7,*], Alberto Migliore[8,*]*

1 Nanofaber S.r.l., Via Anguillarese 301, 00123 Rome, Italy; patrizia.hanieh@nanofaber.com (P.N.H.); noemi.fiaschini@nanofaber.com (N.F.); antonio.rinaldi@nanofaber.com (A.R.);

2 Department of Biochemical Sciences Alessandro Rossi Fanelli, Sapienza University of Rome, Piazzale Aldo Moro 5, 00185 Rome, Italy; anna.scottodabusco@uniroma1.it (A.S.d.); alessia.mariano@uniroma1.it (A.M.);

3 Department of Experimental Medicine, University of Rome Tor Vergata, Via Montpellier 1, Rome, 00133, Italy; valeria.palumbo.25@students.uniroma2.eu (V.P.); manuel.scimeca@uniroma2.it (M.S.);

4 Department of Chemical Science and Technologies, University of Rome Tor Vergata, Via della Ricerca Scientifica 1, 00133 Rome, Italy; venanzi@uniroma2.it (M.V.); francesca.cavalieri@uniroma2.it (F.C.);

5 Interdepartmental Center for Comparative Medicine, Alternative Techniques and Aquaculture (CIMETA), University of Rome Tor Vergata, Via Montpellier 1, Rome, 00133, Italy; bbtcrl01@uniroma2.it (C.A.); mattei@uniroma2.it (M.M.);

6 Department of Chemistry, University of Bari, and CSGI (Center for Colloid and Surface Science), via Orabona 4, 70125 Bari, Italy; luigi.gentile@uniba.it (L.G.);
7 Department of Clinical Sciences and Translational Medicine, University of Rome Tor Vergata, Via Montpellier 1, Rome, 00133, Italy; roberta.bernardini@uniroma2.it (R.B.);
8 San Pietro Fatebenefratelli Hospital, Via Cassia 600, 00189, Rome, Italy; migliore.alberto60@gmail.com (A.M.)

List of the contents

FTIR spectra of Microsponge, free methotrexate, and methotrexate-loaded Microsponge (**Figure S1**); Herschel–Bulkley fitting parameters of rheological measurements for all formulations in PBS and synovial-based media at 25 °C and 37 °C (**Table S1**); frequency sweep rheological experiments of all samples in the presence of synovial fluid (**Figure S2**); estimation of injection force using the Hagen–Poiseuille equation adapted for power-law fluids (**Eq S1**); ELISA quantification of serum IL-1β and TNF-α levels at the end of the in vivo study (**Figure S3**); histological analysis of knee joints stained with Masson's trichrome (**Figure S4**).

FTIR analyses

Fourier-transform infrared (FTIR) spectroscopy was performed using an IRXross spectrophotometer (Shimadzu Corporation, Kyoto, Japan), over the 400–4000 cm^{-1} wavenumber range with 8 cm^{-1} resolution to characterize the solid-state properties of MTX, MSP, and MTX-loaded MSP, and to investigate possible interactions between drug and carrier. Samples were placed directly in the sample compartment, and absorption spectra were recorded. The analysis aimed to determine whether MTX incorporation involved electrostatic, covalent, or purely physical interactions.

To clarify the nature of these interactions, FTIR spectra were recorded for empty MSP, free MTX, and MTX-loaded MSP (**Figure S1**).

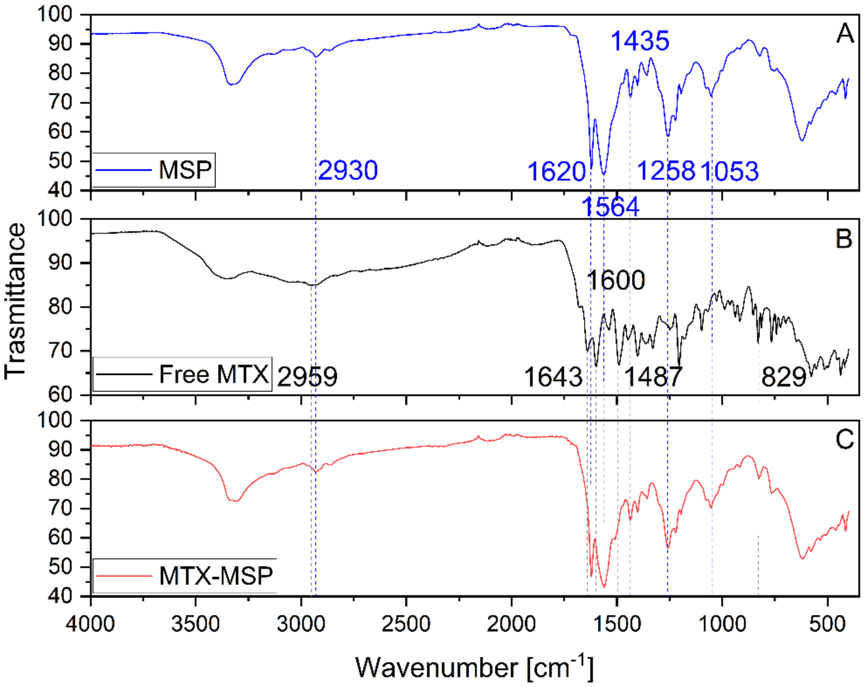

Figure S1. FTIR spectra of Microsponge (Panel A), free methotrexate (Panel B) and methotrexate loaded Microsponge (Panel C).

The FTIR spectrum of empty MSP (Panel A) showed the characteristic vibrational signatures the HA-based polymer network. In particular, a broad band at 3200–3400 cm^{-1} was assigned to ν(O–H) (hydrogen-bonded hydroxyls) with contributions from ν(N–H). The signal at ≈ 2930 cm^{-1} corresponded to $\nu_s(CH_2)$. In the fingerprint region, the intense bands at ≈ 1620 cm^{-1} and ≈ 1564 cm^{-1} were attributed to carboxylate vibrations of HA, namely ν_{as}(COO−) and ν_s(COO−), respectively, while the saccharide-related region displayed contributions around 1030-1040 cm^{-1} mainly associated with ν(C–O) and ν(C–O–C) stretching of the polysaccharide backbone. Overall, these bands confirmed the preserved structural identity of the MSP scaffold.

The FTIR spectrum of free MTX (Panel B) exhibited bands consistent with its functional groups: ν(O–H)/ν(N–H) stretching in the 3400-3200 cm^{-1} region (here observed at ≈ 3450 cm^{-1}), C–H stretching near ≈ 2959 cm^{-1}, and prominent carbonyl-related absorptions in the 1700–1600 cm^{-1} range (here at ≈1643 and ≈1600 cm^{-1}, attributable to ν(C=O) of amide/carboxyl groups coupled with ring modes). Additional features between ≈ 1539 and 1487 cm^{-1} were assigned to δ(N–H) (amide) and aromatic ν(C=C) stretching.

After MTX loading (Panel C), the spectrum largely retained the MSP bands, indicating that the polymer backbone remained unchanged, but it also showed MTX-related contributions, notably the appearance of a shoulder at ≈ 1487 cm^{-1}, assigned to δ(N–H) of MTX (amide-related bending) and/or aromatic modes. Importantly, the main HA carboxylate bands (ν_{as}(COO−) and ν_{s}(COO−)) did not display clear, resolvable shifts beyond spectral resolution, and no new bands attributable to covalent bond formation emerged. Because no band broadening was observed upon MTX loading, the ν(O–H)/ν(N–H) stretching region (3200–3400 cm^{-1}) remains essentially unchanged in shape and width, suggesting that MTX incorporation does not significantly reorganize the HA hydrogen-bonding environment and is consistent with weak/non-specific non-covalent interactions rather than strong complexation. This overall pattern suggests that MTX is incorporated predominantly via non-covalent interactions, most plausibly hydrogen bonding (involving O–H/N–H donors and C=O/COO− acceptors) and electrostatic interactions between ionizable MTX groups and the HA carboxylate moieties, rather than through the formation of new covalent linkages.

Supplementary rheological analysis and Herschel–Bulkley fitting parameters

Table S1 reports the complete Herschel–Bulkley fitting parameters, including yield stress (σ_Y), consistency index (K), and flow behavior index (n), obtained for all formulations dispersed in PBS and synovial-based media at 25 °C and 37 °C. The Herschel–Bulkley model provided

excellent fits for all samples (R^2 > 0.99), confirming yield-pseudoplastic behavior under conditions relevant to intra-articular administration.

Table S1. Rheological parameters for various samples at 25 °C and 37 °C in different media, fitted using the Herschel–Bulkley model.

**Temperature (°C)**	**Sample**	**Medium added**	**σ_γ (mPa)**	**K x 10^{-3}**	***n***	**Reduced Chi-Sqr**	**R-Square (COD)**
25	PBS	-	0	1.03 ± 0.05	1	0.62815	0.99999
	MSP	PBS	99 ± 8	18 ± 5	0.55	557.85375	0.94984
	MTX-MSP		110 ± 7	17 ± 4	0.61	481.34636	0.97077
37	MSP		12 ± 2	0.66 ± 0.05	1	11.853	0.9998
	MTX-MSP		13 ± 2	0.61 ± 0.05	1	15.995	0.99971
	HSF	-	0	373 ± 14	0.50	7930.620	0.99923
	-	HSF+PBS	0	48 ± 1	0.69	161.266	0.99991
	MSP		0	46 ± 1	0.68	227.215	0.99987
	MTX-MSP		7 ± 2	60 ± 1	0.67	69.315	0.99997

The oscillatory rheological data shown in **Figure S2** further support the flow measurements by highlighting the frequency-dependent viscoelastic behavior of the formulations in synovial-based media.

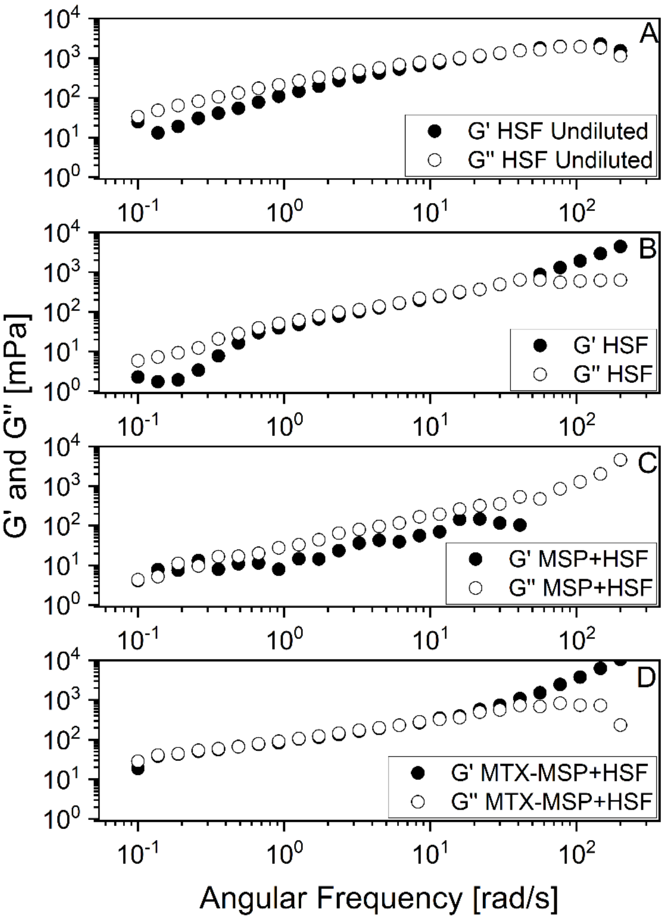

Figure S2. Frequency sweep experiments of all samples in presence of HSF.

Estimation of Injection Force

To evaluate injectability, the injection force was estimated using a generalized form of the Hagen–Poiseuille equation adapted for power-law fluids:

$$F_{fluid} = \left[2^{n+2}\pi^{1-n}LR_s^2\left(\frac{3n+1}{2n+1}\right)^{n-1}\frac{KQ^n}{R^{3n+1}}\right] \quad (1)$$

where L is the needle length (considered 0.016 m, standard 25G needle), R is the internal needle radius (0.00013 m, 25G needle), R_s is the inner syringe radius (0.0026 m), Q is the volumetric

flow rate (1×10^{-7} m^3/s considering 1 mL in 10 s), while K and n are the values obtained from the eq. 1 fitting (the consistency index and the flow behavior index).

Evaluation of Serum Cytokines and Anti-Collagen II Antibodies by ELISA

The quantification of IL-1β levels in rat serum samples was conducted using a commercially available ELISA kit (BMS630, Invitrogen, Thermo Fisher Scientific, Waltham, MA, USA) following the protocol provided by the manufacturer. For the detection of antibodies specific to type II collagen, an ELISA assay was performed as follows: 96-well microplates were coated overnight at 4°C with type II collagen (C1188; Sigma-Aldrich, St. Louis, MO, USA) prepared at a concentration of 2 μg/mL in phosphate buffer solution (pH 9.3), with a coating volume of 100 μL per well. Following coating, plates were washed thrice and blocked for 1 hour at room temperature using phosphate-buffered saline containing 1% bovine serum albumin (PBS-BSA). Serum samples, diluted 1:100, were added to the wells (100 μL per well) and incubated at 37°C for 1 hour. Subsequently, wells were incubated for an additional hour at 37°C with horseradish peroxidase (HRP)-conjugated anti-rat IgG secondary antibody (Invitrogen, Milan, Italy). Optical density (OD) measurements were taken at 495 nm for both collagen-coated and buffer-only coated wells, and specific binding was calculated by subtracting the OD of buffer-coated controls from that of antigen-coated wells. All samples were assayed in duplicate to ensure assay reliability and reproducibility.

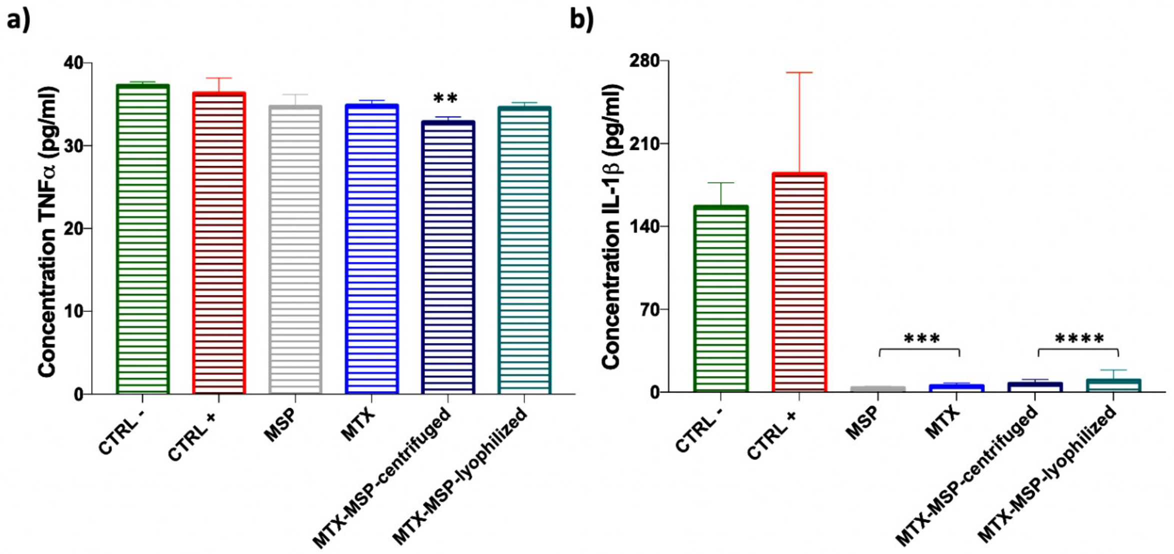

Figure S3. a) Levels of IL-1β cytokine from serum determined by ELISA assay at the end of the experiment; b) Levels of TNF- α cytokine from serum determined by ELISA assay at the end of the experiment. Data are presented as mean ± SD.

* $p < 0.05$; *** $p < 0.001$; **** $p < 0.0001$ compared with positive control.

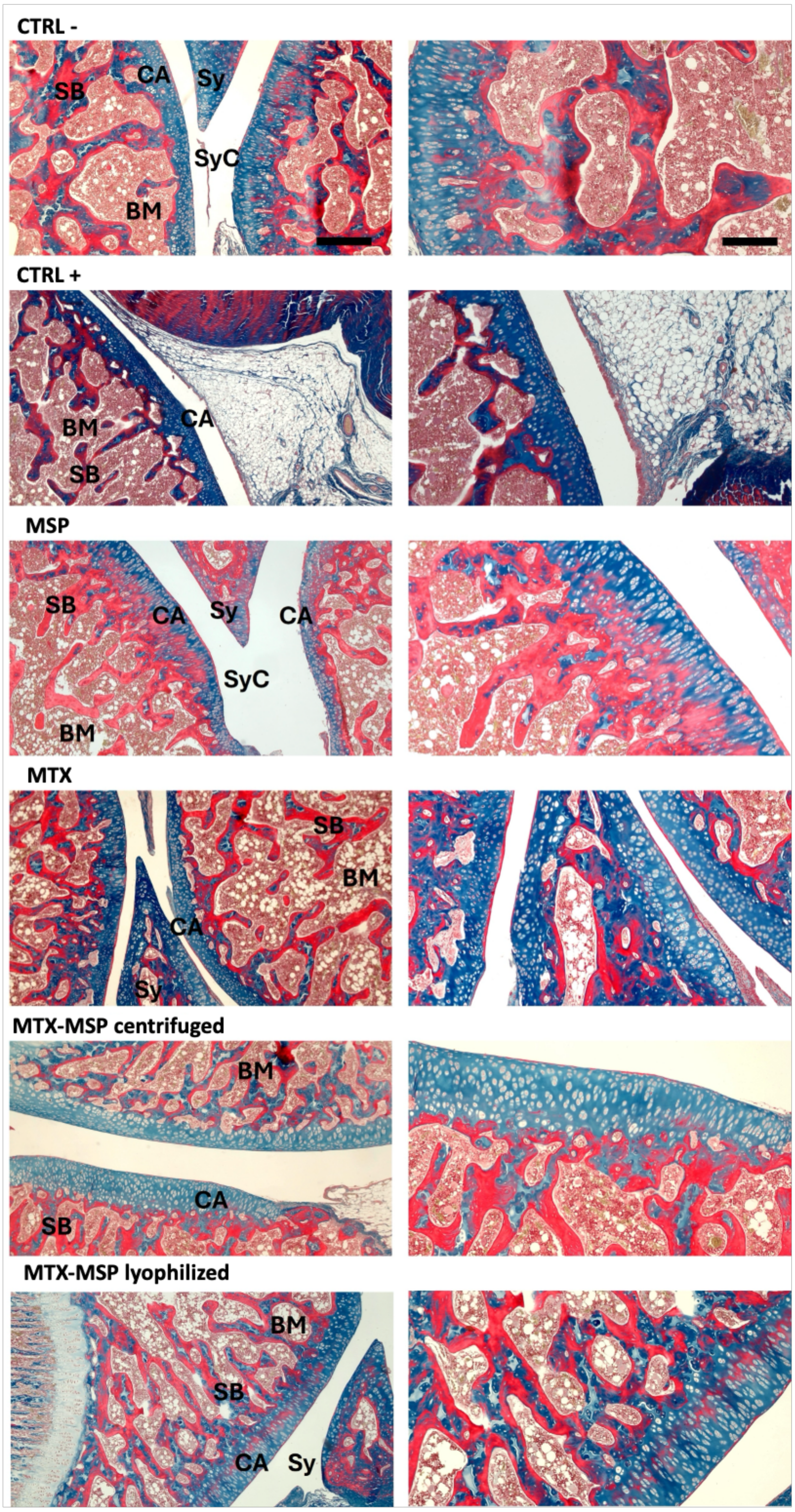

Figure S4. Histological analysis of knee joints stained with MASSON. BM: bone marrow; BV: blood vessel; CA: cartilage; SB: subchondral bone; Sy: synovium; SyC: synovial cavity. Left column: magnification 5×, scale bar 500 µm. Right column: magnification 10×, scale bar 300 µm.
